# Analyses of Tissue Culture Adaptation of Human Herpesvirus-6A by Whole Genome Deep Sequencing Redefines the Reference Sequence and Identifies Virus Entry Complex Changes

**DOI:** 10.3390/v10010016

**Published:** 2017-12-31

**Authors:** Joshua G. Tweedy, Eric Escriva, Maya Topf, Ursula A. Gompels

**Affiliations:** 1 Department of Pathogen Molecular Biology, London School of Hygiene & Tropical Medicine, University of London, London WC1E 7HT, UK; joshua.tweedy@lshtm.ac.uk or joshua.tweedy@cruk.manchester.ac.uk (J.G.T.); eric.escriva@lshtm.ac.uk (E.E.); 2 Institute for Structural and Molecular Biology, Department Biology, Birkbeck College University of London, London WC1E 7HX, UK; m.topf@cryst.bbk.ac.uk

**Keywords:** human herpesvirus, HHV-6, HHV-6A, Roseolovirus, betaherpesvirus, virus entry complex, gH/gL, gB, deep sequencing, reference genome, cell fusion, glycoprotein structure model

## Abstract

Tissue-culture adaptation of viruses can modulate infection. Laboratory passage and bacterial artificial chromosome (BAC)mid cloning of human cytomegalovirus, HCMV, resulted in genomic deletions and rearrangements altering genes encoding the virus entry complex, which affected cellular tropism, virulence, and vaccine development. Here, we analyse these effects on the reference genome for related betaherpesviruses, Roseolovirus, human herpesvirus 6A (HHV-6A) strain U1102. This virus is also naturally “cloned” by germline subtelomeric chromosomal-integration in approximately 1% of human populations, and accurate references are key to understanding pathological relationships between exogenous and endogenous virus. Using whole genome next-generation deep-sequencing Illumina-based methods, we compared the original isolate to tissue-culture passaged and the BACmid-cloned virus. This re-defined the reference genome showing 32 corrections and 5 polymorphisms. Furthermore, minor variant analyses of passaged and BACmid virus identified emerging populations of a further 32 single nucleotide polymorphisms (SNPs) in 10 loci, half non-synonymous indicating cell-culture selection. Analyses of the BAC-virus genome showed deletion of the BAC cassette via loxP recombination removing green fluorescent protein (GFP)-based selection. As shown for HCMV culture effects, select HHV-6A SNPs mapped to genes encoding mediators of virus cellular entry, including virus envelope glycoprotein genes gB and the gH/gL complex. Comparative models suggest stabilisation of the post-fusion conformation. These SNPs are essential to consider in vaccine-design, antimicrobial-resistance, and pathogenesis.

## 1. Introduction

Establishing reference genomes are essential for characterisation of pathogens, their interaction with the host, development of vaccines, and responses to therapies including antimicrobial resistance. Betaherpesviruses are a classic example of effects of laboratory passage of viruses distorting understanding of natural transmission. In this case, betaherpesvirus human cytomegalovirus (HCMV) was originally cultured in human fibroblasts, the reference AD169 strain, with derivation of its genome being the largest at the time [1]. However, increasing evidence showed differences with strains grown in endothelial cells, the cell type relevant for characterising one of the main pathologies of the virus and placental infection causing congenital disease with cytomegalovirus (CMV) the main infectious cause of birth defects, including microcephaly even in the setting of emergent Zika virus. Derivation of the genomic sequence of early passage clinical strains showed that an almost 20kb deletion occurred in the AD169 genome [2]. Subsequent extensive analyses, using next generation sequencing technologies, have shown that clinical isolates, which are not cultured, do not have this deletion. Furthermore, genes located in the “deleted” region encoded essential elements dictating the cellular tropism of the virus. Alternate gH/gL complexes were established as main components of cellular tropism and fusogenic entry of the virus into cells. The trimer, gH/gL/gO, related to fibroblastic entry of cells while the competing pentamer form gH/gL/UL128/UL130/UL131 dictated endothelial, leukocyte, and epithelial cell entry [3]. The pentameric components were encoded by genes in the “deleted” region of the genomes of laboratory-passaged virus. bacterial artificial chromosome (BAC)mid “repair” of these genes rescued infection defects, but also could be mutagenic themselves, if there were single nucleotide polymorphisms (SNPs) affecting efficiency of tissue culture replication [4]. The resulting complexity of these components are essential to vaccine design and generation of a neutralising antibody, even to new vaccine vectors directed at other infections and modulating cellular immunity [5,6,7]. These could never have been identified without the derivation of the actual wild type genomes involved in natural transmission.

Similar analyses of polymorphisms in the reference human betaherpesvirus 6, HHV-6A, have not yet been conducted. However, isolated reports indicate homologous regions are targeted and may also occur in virus replicated in vivo. HHV-6A also encodes a gH/gL complex central to infectious entry, as well as alternate multimeric complexes affecting cellular tropism; these are a related trimeric complex gH/gL/gO and a quadrameric complex gH/gL/gQ1/gQ2 [8,9,10,11]. The *gQ1* and *gQ2* genes are in the homologous pathogenicity region to that in HCMV. Polymorphisms in gQ2 have similarly been detected in tissue culture passaged virus as well as in a BACmid clone of human herpesvirus 6A (HHV-6A) strain U1102 [12]. Furthermore, there is a positional homologue of HCMV chemokine like molecules UL128/130/131, which is HHV-6A chemokine U83A [13]. Polymorphisms in HHV-6 strain U1102 virus passaged in vitro as well as in vivo strains have been reported, which affect translated reading frames of this gene, alternating between an extended encoded mature secreted chemokine and a non-secreted version [13,14]. In addition, a few corrections have been reported, including a CG inversion in U83A [15], GG insertions in DR6 [16], and nucleotide insertion in U86 [17]. Two distinct but closely related genomes of HHV-6A strain GS have been reported (HHV-6A GS KJ123690.1 and KC465951.1), indicating potential for virus population polymorphisms; however, initial reports of this strain as a human B-lymphotropic virus (HBLV) describes isolation from six patients, therefore, there could be mixtures [18]. A systematic analysis has not been undertaken to evaluate in the type reference genome of HHV-6, HHV-6A strain U1102, populations of viruses generated from tissue culture passage or present from in vivo polymorphisms. This is essential to understanding the pathogenicity of HHV-6 as a HCMV.

Human herpesviruses cause a range of pathologies from oral diseases, incurable genital infections, to cancers. HHV-6A is linked with neurological and cardiac disorders in immune suppressed or naive patients [19], including fatal infant myocarditis [20,21]. Roseoloviruses, mainly HHV-6A and HHV-6B, are unique among human herpesviruses in integrating their genome in the human germline at the chromosomal telomere, termed CiHHV6A and CiHHV6B. This affects approximately 1% of people worldwide—there are upwards of 70 million people at risk of virus reactivation in every cell, and recent studies show links with impaired infant neurodevelopment and cardiac disease [14,22,23,24,25]. Genomic studies show the reference HHV-6A distinct from CiHHV6A, but the full extent of polymorphisms in the reference strain are required to understand relationships with CiHHV6A in order to interpret clinical conditions and potential treatment for virus reactivation.

Our studies using deep sequencing showed we could distinguish between known circulating HHV-6A viruses and those that are integrated in our genomes, CiHHV-6A [14,24]. With next generation sequencing technologies, we could amplify specifically the virus sequences separate from the human genome in order to characterise their differences. We combined this method with whole genome deep sequencing, where the short reads map to a reference template genome and are amplified thousands of times, which enables rare mutations in a population to be detected. We can significantly detect these “minor variants” down to 1% in a mixture and have used this method to detect minor variants from virus superinfection. Viruses superinfecting patients with integrated genomes were detected as 1–30% mixtures by using SNPs representative of superinfecting virus genomes [24].

This methodology was applied here to redefine the Roseolovirus type reference, HHV-6A strain U1102, using an early isolate and to characterise population polymorphisms and minor variants arising from effects of tissue culture passage of virus and in BACmid virus reconstruction. This was done in order to test whether the same mutagenic effects on the virus entry complex as shown for HCMV were also affected in HHV-6A. We further validate these polymorphisms by reference to in vivo virus “cloned” by integration in the germline. This not only defines the reference but also the changes critical to virus spread during cell culture that are relevant to general understanding of infection mechanisms.

## 2. Materials and Methods

### 2.1. Cells and Viruses

HHV-6A strain U1102 low passage from the original isolate, P3, was grown in JJhan cells as described [26]. The continuous cultured virus, termed pU1102 here, was grown on HSB2 cells (Kindly donated by B. Prusty from the University of Wurzberg, Würzburg, Germany) and a virus reconstructed from cloned BACmid version BAC-U1102 (kindly provided by Y. Mori, Kobe University, Japan and cultured by B. Prusty, University of Wurzberg, Würzburg, Germany) [12,27] were identically passaged in HSB2 cells, as described, for four serial weekly passages [28,29]. CiHHV-6A strains 5055, 2284, and 5814 were extracted from DNA from patients as described in [24].

### 2.2. Illumina Deep Sequencing

The five virus-infected cell DNA samples were extracted (Qiagen, Hilden, Germany) and CiHHV6A DNAs were prepared for target enrichment amplification and deep sequencing as described. These were HHV-6A U1102, bU1102, BAC-U1102, K21-HHV-6A-pU1102, and K21 BAC-U1102. For the specific amplification, 36 overlapping primer pairs were used which amplified overlapping PCR products across the complete genome as described, with all predicted sizes amplified (see primers in Table 1). These were separated by agarose gel electrophoresis, and the purified products pooled in equal quantities as measured using Qubit. Libraries were prepared by sonicating the DNA using Covaris, followed by purification and random PRC amplification using adaptor tags from the NEBNext DNA library kit. Tagged samples were run on a MiSeq and the resulting raw FASTQ files analysed after quality assessment and removal of adaptor tags and primer sequences as we described previously [24].

The reads were trimmed using trimmomatic with quality scores applied, then assembled using Samtools and Burrow-Wheeler Aligner (BWA)-mem alignment algorithm by mapping to reference genomes using the HHV-6A U1102 reference genome (updated from denovo assemblies) as described [24]. Coverage and qualities were assessed using BAC-U1102 complete coverage as a template. Contigs were ordered using the reference genome with manual adjustment using Artemis. Variant calling was applied with both Genome Analysis Toolkit (GATK) UnifiedGenotyper and SAMtools mpileup using BCFtools, vcfutils varFilter pipeline, quality scores >25, and minimum read depths of 10 for base call accuracy [24,29].

The rapid annotations tool Rapid Annotation Transfer Tool (RATT) was used to transfer annotations and syntenic coordinates from the reference genome [30] (HHV-6 U1102 NC_001664). All SNPs were compiled and compared in an excel database using a custom python script. Tables were tabulated for corrections and polymorphisms. All SNPs from K21 treated samples were compared using the unexposed HHV-6A pU1102 and BAC-U1102 samples as template. Average coverage was 2000–10,000 reads depth with a sensitivity variant cut-off of 1%. All results using target amplification were compared to those generated via SureSelect (Agilent, Santa Clara, CA, USA) enrichment and de novo assembly as described [31], and were in agreement with no evidence for gaps. The re-defined reference genome is deposited for European Bioinformatics Institute (EBI) (updated to the original deposition X83413) and Genbank (reference genome NC_001664).

### 2.3. Molecular Models

Comparative models were generated for HCMV gB from strain Merlin (UniProtKB/Swiss-Prot: F5HB53) and for HHV-6A U1102 gB from wt and passaged strains in the post-fusion conformation using Modeller9v7 [32]. The modelling was based on multiple templates from gB HCMV strain AD169 (PDB ID: 5cxf) and strain Towne (PDB ID: 4osn and 5c6t), gB Epstein-Barr virus (EBV) strain B95-8 (PDB ID: 3fvc), and gB Herpes Simplex virus (HSV)-1, strain KOS (PDB ID: 2gum and 3nwa). Closest alignments were shown with HCMV gB, 45% identity, 65% similarity (meta method M-Coffee, see Supplymentary Materials Figures S1–3). The models cover the ectodomain, residues 38–579, and domains I, II, III, and IV. The passage mutant, Thr193Ala, was modelled using swapaa command in Chimera [32]. The models of the HHV-6A gB trimer for the domain I of wt, and passage mutant, Thr193Ala, gB were obtained by structural alignments with the structure of the gB HCMV trimer strain AD169 (PDB ID: 5cxf) with each of its subunits. The free energy predictions of the Thr193Ala mutation on the subunit-subunit interaction stability was done using web server mCSM [33], which predicts stability changes of a wide range of mutations from graph-based signatures encoding distance patterns between atoms.

## 3. Results

### 3.1. Deep Sequence Analyses of Reference HHV-6A Strain U1102 Shows Corrections

Illumina deep sequencing was conducted deriving HHV-6A strain U1102 genomes between 2000–10,000 times per group. These included three versions of HHV-6A strain U1102: low passage (P3), continuous cell passage, and BAC reconstructed virus. In addition, sequences were derived for the continuous passage virus (pU1102) and the BAC virus (BAC-U1102). The Miseq run was derived at the same time as resequencing CiHHV6A strains 2284, 5055, and 5814 used to confirm SNPs. Consensus sequences were determined and minor variants tabulated and quantified.

The first analyses compared the NCBI Genbank reference genome HHV-6A strain U1102 genome to the deep sequenced early passage of the original isolate of strain U1102. In order to identify corrections to this reference sequence, all polymorphisms subsequently identified in the passaged strains were removed. Table 2 identifies 32 corrections to the reference sequence, including six previous citations, and one indel (U10). Nine of these included non-synonymous coding changes with five previously described changes (Table 2).

Newly identified changes were in genes U40, U57 (major capsid protein), U58, and U100 (gQ1 glycoprotein). It is possible these were mixtures of minor variants, below <1%, not detected in the original sequence. Therefore, these were also compared to genomes from cultured passaged virus, as well as from artificial cloned virus genomes using BACmid technology or naturally cloned virus genome present in the germline of people with chromosomally integrated virus genomes, CiHHV6. These were all re-sequenced at the same Illumina run, and none of these genomes showed evidence for these SNPs screened down to 1% sensitivity by deep sequencing.

Only five further SNPs were identified from a potential mixed original population. Two were previously described in U83 and gQ2 [13,14,27], but were not detected here down to 1% sensitivity. A further three SNPs comprised 88–97% of the population, but in non-coding repetitive sequences in the direct repeat (DR) region and the left end of the main unique region. The DR indels were also identified in the integrated genomes suggesting natural variants. This confirmed 32 corrections and 5 population variants out of 152,921 bp in the genome derived in the original isolate. Therefore, the original Sanger sequencing of the virus isolate was 99.98% accurate, which was not possible with Illumina next generation consensus sequencing methods, but resolved here with additional deep sequencing and analyses of population variants.

### 3.2. Changes with BACmid Cloning and Reconstruction

Having established a new reference genome sequence for HHV-6A strain U1102, this was then compared to the whole genome sequence of BACmid cloned virus. This had been cloned as BACmid using a BACmid cassette inserted in-between genes U53 and U54 [27], then the virus was reconstructed from tissue culture passage as described [28].

In total, 16 SNPs were identified including two indels (one in the U86 immendiate early 2 (IE2) repeat and one in an intron of U100 gQ1). Six of those were non-synonymous changes in structural coding genes U31 tegument, U33 capsid, U39 gB, U50 capsid, U86 IE2, and U100 gQ2 (Table 3). The SNP in gQ2 was distinct from a polymorphism previously reported which resulted in a premature stop codon giving a truncated gQ2 [12]. Instead, it was a single amino acid substitution (Table 3).

**Figure 1:**
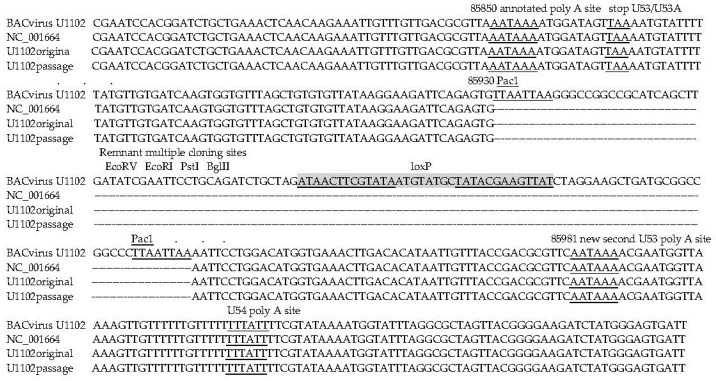
Passaged bacterial artificial chromosome (BAC)-virus deletes the green fluorescent protein (GFP) cassette. In the BAC virus, the GFP cassette disrupts the U53/U53a transcriptional control elements, and after tissue culture passage it is deleted. Shaded and underlined are palindrome1, palindrome 2, and LoxP sites; note the insertion is in an end repaired EcoRI site. Underlined are the original and new poly A regulatory sequences disrupted by the BAC cassette.

The only major rearrangement observed was at the site of the BAC cassette insertion. This cassette is derived from transfer cassette pHA2 [36] and includes loxP sites bounding a gpt, BAC related and green fluorescent protein (GFP) genes. This was rearranged, showing all the genes deleted with only one loxP site remaining indicative of recombination at this site. Moreover, the site of BAC insertion interrupts a newly identified distal poly A site for the essential U53/U53A proteinase/capsid scaffold genes (Figure 1). Therefore, mutagenic effects related to BAC reconstruction are demonstrated here.

### 3.3. Emerging Variant Genome Populations Generated after Tissue Culture Passage

Next, using deep sequencing, the new reference HHV-6A strain U1102 genome sequence was compared to genomes derived from virus, which had been continually passaged (four passages) as infected cells. The results showed that the passaged virus consisted now of an expanded population of minor variants with the now expanded major variants emerging to dominate (Table 3). Interestingly, the variants, which arose by culture, included all the five non-synonymous SNPs in structural genes identified in the BAC virus reconstructions. In addition, there were three further non-synonymous mutations in replication genes. In total, there were 24 SNPs, including six indels, all non-coding, four in DR, and one in the left end repeats of the Unique region, one in the R2 repeat and one in the intron within U100 gQ1. The 24 SNPs ranged from 52% to 99% of the mixed population.

Almost half of the SNPs, 11/24, were shared with the SNPs in the BACmid population of reconstructed virus genomes, therefore, indicating selection for tissue culture. These included the non-synonymous coding changes in U31, U33, U39 gB, U50 capsid, and U100 gQ2, which are mainly structural or envelope genes affecting infectivity. The new SNPs in this population included non-synonymous coding changes in U2, U41 DNA binding protein, U73 origin binding protein, U86 IE2, and three in U95. There were also two SNPs within the origin for replication. Of these, only three have been reported in another strain (GS). This strain is also a mixture from six original patients; two SNPs were reported for GS-KJ123690.1 (U41 and origin of replication, ORI, repeat) and two SNPs for GS-KC465951.1 (U50 capsid and the ORI repeat). The other non-coding changes are uniquely reported here, U2, U73 origin binding protein, U86 IE2, and the three in U95, which are mainly non-structural genes affecting replication. These may represent low-level variants present in the original virus isolate expanded here or the effects of adaptation to tissue culture and improved replication. Notably, all the BACmid virus SNP coding changes were present as variants in the continually passaged virus population, identifying these as most likely tissue culture adaptations.

### 3.4. Virus Envelope Genes Mutations from Tissue Culture Passage Affect Fusion Complex

Both the BACmid and cultured virus accumulated mutations in envelope glycoproteins of the entry complex, which mediate cellular fusion. This included glycoprotein gB, which has been characterised as a fusogen conserved in herpesvirus and also gQ1 and gQ2 which are part of the gH/gL/gQ1/gQ2 complex. Like gB, the gH/gL complexes are also conserved in herpesviruses, but there are additional trimer or quadrameric versions in Roseolovirus. The gH/gL/gQ1/gQ2 components are specific to Roseoloviruses; this complex interacts with a cellular receptor CD46, and is a target for a neutralising antibody, which inhibits cellular spread [11,41,42]. These genes are positional homologues of the UL128/130/131 pentameric complexes in HCMV which are also mutated by virus passage in cell culture [43]. The remaining complex gH/gL/gO is unaltered from tissue culture of both viruses. Although at present there is not a structural model for gQ1 or gQ2, the complex interacting with gH/gL can form the fusion complex as for other herpesviruses [12].

**Figure 2:**
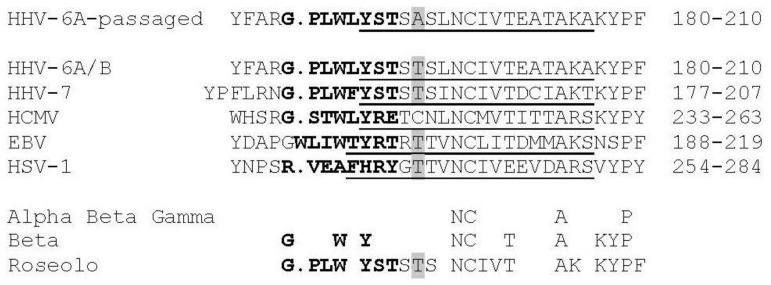
Human herpesvirus gB fusion loop 2, FL2, region sequence comparisons show unique SNP substitution in passaged HHV-6A virus. Comparison of the FL2 (in bold) and associated sequences of HHV-6A gB (residues 184–191 in FL2 in bold) from passaged virus (and cultured BAC virus) compared to gB from 51 clinical samples of HHV-6A/B [45], CiHHV-6A/B [14,24], and 50 clinical samples of HHV-7 gB [46] demonstrating the Thr193Ala mutation is only found in passaged virus. This is compared to reference sequences from alpha, beta, and gamma herpesvirus gB where the structure has been determined, as shown for HCMV gB structure, identifying the beta sheet 12 in domain I [40]. Alignments were made using Clustal as described [40] with gB from HCMV (shown for strains Merlin, Towne, and TB40E and 74 clinical strains; strain AD169 and six clinical strains have Val252Leu), EBV (strain B958), and HSV-1 (strain KOS) (UniProtKB P06473, Q777B0, P06437, respectively). The FL2 in HCMV, EBV, and HSV-1 gB are in bold, and predicted FL2 for HHV-6A/B and HHV-7 are similarly indicated. FL2 in HHV-6A/B gB is amino acids 184–191 and in HCMV, strain Merlin, 237–244. This is adjacent to a conserved beta sheet shown in the structures of EBV, HSV, and cytomegalovirus (CMV) gB as underlined, and that predicted for HHV-6A/B and HHV-7 indicated (See Methods). The Thr193Ala passage SNP is shaded in the FL2 adjacent region, disrupting the polar patch and increasing hydrophobicity. This Thr is conserved in HHV-6A/B, HHV-7, EBV, and HSV.

**Figure 3:**
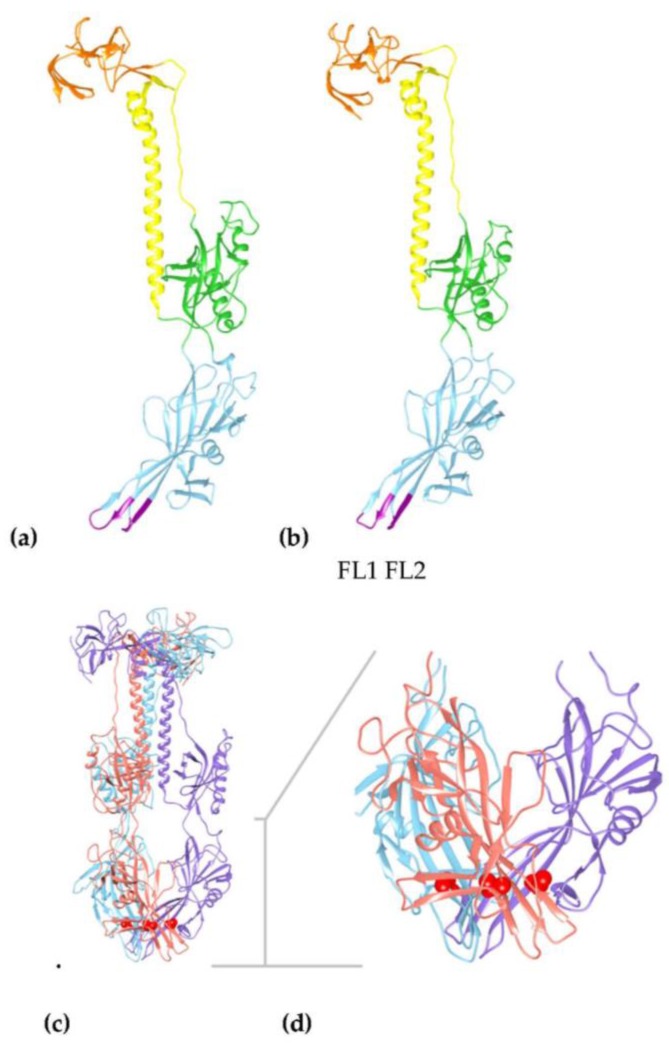
Structural models of HHV-6A gB show conservation with HCMV gB structure identifying the passaged virus has a mutation in the fusion associated domain I affecting the trimer interface. (**a**) Model of the HCMV reference strain Merlin (ectodomain in residues 91–642 from a total of 907), alignment with corresponding region of HHV-6A gB is shown in the Figures S1–3. (**b**) Model of HHV-6A gB ectodomain, in the corresponding residues 38 to 579 (total 839 residues) using multiple templates of pdb (RCSB protein database) folds from gB HCMV strain AD169 (PDB ID: 5cxf) and strain Towne (PDB IDs: 4osn, 5c6t), gB EBV strain B95-8 (PDB ID: 3fvc), and gB HSV-1 strain KOS (PDB IDs: 2gum and 3nwa). Using the structure of most closely related HCMV gB, as a model, shows Domains I (blue), II (green), III (yellow), and IV (orange) in the post-fusion conformation. Domain I (blue) shows conserved beta sheets connected by the two fusion loops, FL1 and FL2, at the base of the model (purple). (**c**) Longitudinal view of the predicted trimer of HHV-6A gB showing the Thr193 position in red within three subunits, indicated in purple, blue, and orange. (**d**) Domain I (residues 79–291) is enlarged as indicated by the grey T bar, showing the position of Thr193, in red, in the beta sheet at the trimer interfaces with the fusion loops FL1 (residues 100–105) and FL2 (residues 184–191) exposed at the base of the structure.

There are crystal structures available for homologous gB molecules from HCMV, HSV, and EBV (see Material and Methods) in the post-fusion conformation [40,44]. Using these structures, we modelled the tertiary structure of HHV-6A U1102 gB affected by the mutation Thr193Ala from cell passage (Table 3, Figure 2 and Figure 3). This mutation is not found in any homologous gB sequence analysed to date (BLAST Genbank scans). Alignments show that this mutation is located in the Beta 12 pleated sheet of Domain I (DI) of gB (based on the HCMV homolog) in the vicinity of the second fusion loop, FL2 (residues 184–191; Figure 2 and see Figures S1–3). Thr193 aligns to the unpaired Cys246 residue in HCMV gB. Further, DI not only is the fusion domain, but also the site suggested of interaction with gH/gL in potential triggering of cell fusion [40]. Thr193 is conserved in HSV and EBV gB next to a conserved motif central to the beta sheet (Figure 2 and Figure 3).

The passage mutation Thr193Ala is unlikely to affect the beta sheet prediction adjacent to FL2 (beta sheet 12 in HCMV domain I), but the reduced polar residues away from FL2 and increased hydrophobicity may promote membrane association via the Domain I structure as the mutation is within the FL2 adjacent beta-strand (Figure 2 and Figure 3). However, this residue seems to be interacting with residues at the trimer interfaces in Domain I (Leu93, Phe95, Ala147, Asp151, Thr191, and Leu195) (Figure 3 and Figure S2). Alternatively, the Thr193Ala mutation may modify the angle of inclination of the beta-sheet, increasing the interaction between the fusion loops and the membrane, although more likely stabilising the subunit interactions in the hydrophobic pocket as shown by increases in stability predictions (Figure S2).

## 4. Discussion

We used whole genome deep sequencing to re-define the reference genome of the Roseolovirus betaherpesvirus with unprecedented precision and further demonstrated emerging populations of minor variants from the effects of tissue culture passaging of virus and BACmid reconstructed virus. The results have implications for understanding cellular spread as well as clinical interpretation, analyses of drug resistance, and vaccine designs to inhibit virus entry. We analysed the original HHV-6A U1102 isolate in comparison to cell culture adapted virus and cell culture reconstructed BACmid virus. We also compared it to natural “clones” of HHV-6A in the integrated CiHHV-6A genomes we previously derived and re-sequenced here. Specifically, 32 SNP corrections were made to the reference genome sequence, which included nine corrections previously reported (Table 2) giving an overall original accuracy of 99.98%, higher than that of consensus sequencing by Illumina which is 99%. However, combined with deep sequencing of the original isolate, this allowed enhanced resolution as well as definition of emerging minor variants in the cell culture passaged virus, and many of these dominated the mixed population and were detected as “major” variants. There were mixtures of variants in both the passaged HHV-6A U1102 and BAC-U1102 virus stocks with 34–99% mixtures compared to the redefined reference genome. In the passaged stocks, HHV-6A pU1102 and BAC-U1102, there were 10 and 6 genes with non-synonymous SNPs, respectively. Therefore, both the HHV-6 pU1102 and BAC-HHV-6-U1102 stock viruses had polymorphic mixtures. This also is the first derivation of the genome of the reconstructed BAC-HHV-6-U1102 and the polymorphisms shown here comprise a subset of those in the passaged virus, identifying cell culture specific effects.

The passaged stocks were shown comprised of complex polymorphic mixtures, with SNPs averaging 50% mixtures with the wild type reference virus genome. We used screens to the 85 defined coding genes as described for both virus and integrated genomes [24,26]. There were SNP mixtures in both the passaged virus stock as well as the cloned BACmid virus stocks. The passaged virus had 24 SNPs identified.

Mixtures and variants of the strains of HHV-6A as well as related HHV-6B have been described previously. In these cases, the dominant SNP created the consensus sequence. The initial virus isolates from patients were not clonal and since the virus is cell associated, the virus strains were not clone purified previously. For example, there are at least two consensus genomes for HHV-6A strain GS [47] (HHV-6A GS KJ123,690.1 and KC465,951.1).

Using BACmid technology, which “clones” the virus genome using a bacterial artificial chromosome sequence, BAC, with plasmid elements, this should clonally purify the virus stock. However, recovered virus has to replicate in cell culture, and studies from another herpesvirus, HCMV, show that complex mixtures of SNPs can accumulate during selection for replication in tissue culture. In the case of the only described HHV-6A BACmid, BAC-U1102, we also show here there are SNPs compared to the wild type genome. Some could be present in the virus stock used to generate this BAC, but the mixtures shown here also indicate there is some selection during growth in culture.

In the original description of the derivation of BAC HHV-6A U1102, four clones showed varying BAMH1 restriction endonuclease digestion profiles, indicating either mixtures present in the parent U1102 stock used or mutagenesis during the derivation of the BACmid clones. Notably, only one of these clones, G1, was able to reconstitute virus after transfection and co-culture with cord blood mononuclear cells (CBMC). This BAMH1 restriction enzyme profile most closely resembles that reported for the original isolate [19,48]. However, reports using this BAC cloned virus have observed low replication efficiency, so it is possible that the SNPs identified here contribute to this phenotype. It is notable that there are fewer SNPs in the parent BACmid stock, 16 SNPs compared to 24 in the passaged HHV-6 U1102 stock, which could indicate that continuous passage of virus in culture permits accumulation of further SNPs.

Rearrangements in the BACmid may also affect virus replication efficiency. The BAC virus genomes had deletion of the GFP gene. Only the recombined loxP site and associated sequences from the original vectors were maintained in both the parent BAC and the K21 treated BAC virus. Although this could be from homologous recombination mediated by the host, the efficient rearrangement in the entire population suggests a virus gene product is mediating this event. This would confound any selection process dependent on GFP, since mutations or rearrangements would have to occur to reduce this activity. Since GFP expression has been used to characterise genome integration, it is likely that selection for GFP may be artificially enhancing integration as demonstrated [49,50,51]. Analyses showed that the GFP insertion disrupts utilisation of the downstream polyadenylation site, which could be a more efficient transcript. The upstream polyadenylation signal is prior to the stop codon, and with cleavage 10–30 nucleotides downstream, this could result in transcript cleavage and polyadenylation before the stop codon, resulting in an unstable transcript. This could affect the interpretation of studies using BACmids to study replication or integrations. For example, if GFP is selected, this could select for virus with inefficient virus replication, and promote virus genome integration. Further, the remaining bacterial origin sequences may affect replication and integration. The GFP insertion may interrupt the alternate downstream polyadenylation site, giving inefficient expression of the essential scaffold protein p53A and lower virions. Therefore, use of this BACmid for replication or integration studies can give confounding results and either new non-rearranging BACmid or different approaches are required to understand these pathways.

The SNPs in BACmid reconstructed virus are shared with those in the passaged virus, showing effects of cell culture passage, and all are in structural genes. In comparison, the virus-passaged genomes had only three additional SNPs, all related to replication, in the origin binding protein, and the immediate early genes IE2 and U95 “IE3”. There were five shared structural changes, two in capsid and tegument genes, U31 and U33, with the remaining three in envelope glycoprotein genes affecting virus cellular entry by cell fusion. These included gB and the gH/gL complex components gQ1 and gQ2. These may be culture adapted changes for improved growth in leukemic cell lines JJhan, SupT1, and HSB2, which may affect the selection. In contrast, clinical isolates were prepared on primary cord blood stem cells. The population of BACmid SNPs were in structural genes and the virus envelope and these, including gB Thr193Ala, were all contained in the passaged population of SNPs, which also included SNPs in essential replication genes. Of particular relevance, we showed treatment of BAC virus with a novel antiviral affected membranes, reverted the population to wild type, and eliminated the cell culture passage SNPs in the envelope glycoproteins [29].

In HCMV, cell culture causes changes in the virus entry complex, specifically in components of the pentameric complex of gH/gL, in UL128, UL130, and UL131. In HHV-6A, the genes in the homologous genomic position also encode a fusion complex alternate to the trimeric gH/gL/gO. This is the quadrameric complex of gH/gL including components gQ1 and gQ2. In addition, there is a chemokine gene, a virokine, U83A, which is the positional homologue of UL128, but appears to independently direct cellular recruitment for dissemination or immune evasion. There is polymeric DNA editing polymorphism in this gene disrupting expression, the full-length gene is only rarely identified in culture, but has been identified in “cloned” integrated genomes [14]. Therefore, effects of cell passage of both virus and BAC virus show similarities to the mutations arising in HCMV after cell culture passage. The gH/gL/gQ1/gQ2 complex binds the receptor CD46, and it appears that in HHV-6A cell culture, passage favours mutation of the alternate complex to gH/gL/gO, which may also distort cellular tropism and immune responses as shown for CMV. Distinct from HCMV, we show virus and BAC-virus laboratory passage also results in mutation of the fusogen gB, in addition to the fusion regulator, the gH/gL complexes.

HHV-6A gB is closely related to HCMV gB, which enabled modelling of the ectodomain in the post-fusion conformation with high confidence. This demonstrated the mutation, Thr193Ala, was in Domain I, which mediates cell fusion, specifically in proximity to the fusion loop 2 (FL2) adjacent region within a conserved beta sheet. Interestingly, recent studies identified a cell culture induced mutation in the Ebola virus GP_1,2_ glycoprotein, which increases virus cellular spread. This is also in the fusion loop adjacent region, a Thr544Ile mutation, which also increases hydrophobicity [52]. The modelling of HHV-6 gB mutation showed that the mutation is located at the predicted trimer interface region, which may increase the stability of the post-fusion conformation through increased hydrophobicity (Figure S2). Interestingly, in treatment with a membrane directed antiviral, the treated virus no longer retained the gB mutation, and comparisons with the mutated passaged virus in immune precipitation analyses did show increased stability of gB [28,29].

The cell culture passage mutation in the FL2 proximal region is within domain I, the site of effects by gH/gL based on experimental results, although no stable intermediate has been identified [40]. In HSV gH, the N-terminal region proposed to affect gB overlaps with the site of gL interaction and a complex conformational epitope, interacting with a neutralising LP11 antibody [53,54]. A similar structure has been demonstrated at the N-terminus of HHV-6A gH, with mutational analyses demonstrating that a conformational change between interactions of the N and C terminal domains is required for cell fusion [8]. Campadelli-Fiume and colleagues have demonstrated that for the HSV entry complex, gL removal is required for fusion [55]. Therefore, the cell passage mutation in the HHV-6A FL2 proximal region in gB domain I could potentially affect this cell fusion regulation favouring cell-associated virus spread. Recent studies on the gB fusion loops indicate the FL2 proximal region may influence the angle of membrane association [56].

Taken together, our overall studies have redefined the reference genome and identified SNPs associated with cell culture and BAC virus passage, which can affect virus infection by cell fusion as similarly demonstrated in the related betaherpesvirus HCMV. Characterisation of host interactions, genome integration, immune interactions, and antiviral resistance all need to take account of the effects of a diverse mixed genome population present in laboratory-passaged virus and highlight the components of the virus entry complex affecting cellular spread.

## 5. Conclusions

The reference virus genome was re-defined by deep sequencing of the early clinical isolate. Tissue culture passaged virus accumulated a population of genetic variants in genes affecting infection and replication. Virus culture of BACmid virus accumulated a subset of these SNPs in genes affecting infection, including envelope genes gB and gQ1 and gQ2 of the gH/gL complex. Efforts to study virus properties need to focus on the newly defined reference strain. New BACs or different culturing protocols may be required to do this. Additional study of these cell associated mutations from BAC and passaged virus would provide further understanding of cell associated infection and regulation of virus production versus integration.

## Figures and Tables

**Table 1:** Long range PCR genome amplicon primers.

Amplicon	Forward Primer (5′-3′)	Reverse Primer (5′-3′)	Size (bp)
DR1	ACAACCGCATCTTCTTCAG	ATGAACGGAGATCTGGGAG	1750
DR1DR6	CCTCATCTGTTATTCCCTTCC	ACTCACCGCAGTCTTGAAC	2872
DR6	TCCAAACTGTACGGCTATG	TGGCGTCTAATCCAGGTAC	1858
DR_UL	CATGAGAGGACACTGGACGTACTC	GACATCTGTGGACCATGCA	2339
0203	ACCATGTGCGGAGAAAGTTG	ACGAGAATGAATATGCCGATG	2174
0306	ACTCCTATCCGTCTAAACTGCT	ATTTCTCACGCCGGTATTC	4417
0611	ATGCCAAATCTAGCTGCTG	GAATCGGAGGAAGCTAACGT	4757
1114	TCTTCAGGTGTCTCAACCTTC	TGATGATCTGCTGTTGGAAC	5737
1419	TTGAGATGCAGAGCCTCTC	ACGTGGTGGATAATTCTTCAG	5370
1923	CTACATCATCGGTTGCCATA	TACAGAAGTTTCGGCAACTG	5216
2328	TAGTCGCTGGATGTCGATAC	GAGGAATTGTGGACAACGCT	4721
2830	CATCCAACTCCTTGTCCGT	TCGTTCGACGTGTCTGAC	5753
3031	GACACTGTCCAACGCATCT	CTCTTGGGCATAAGTCCAGTA	6552
3134	ACAGAGCGACACCCAATATTC	AGCTGTGTCGATGTCTTGCTA	2787
3439	TGCATTCTCTCACAGCATG	TGTTTCCTTATGCCACCAATC	6309
3941	TGAGGCATGAGACTCAGAAG	AGCGGCATCCATAATGCTTA	4796
4142	AGCATCGTTTGAAGACACC	GAGTTGTTGTTCGCTGGTCT	2810
4142INT	TTGTTTCAGGCCAGCATC	ATACCGAAAACTCAGGCAAC	2983
4245	AGACCGCATTCGTATCGCT	CACGATCAAGTTCGAATCGTA	5092
4549	GAGTGCTCGGATAAGTTCATT	ACGATACAATCCGGAATGC	5922
4953	TGGAACTCGGAATTGTTTCT	GTTTGAGACTGTATCTGCGCA	4282
5357	TATGGTGAAGAACCGTCG	TCCGCAGATAACAGAATGG	4707
5758	CAGTGCCATCTGTTATGAATAG	AGTCGCAGTAAGGTCCACGT	6792
5864	TGCTGAAATTACCTCAGTGAG	CCATTCAGTATAATGCATCC	4229
6469	CTGTATCAATATCAAGGCGG	ACCACTGAATACGAACGCTC	5111
6974	GCGATTGTTCTGCGATAGAG	CTTATATCCGAGCCTTGCAGT	6472
7476	GAGAACATGCTAGACAATTGG	CAAGAACTGCGACTCAATCC	2668
7679	GTTGCTAGTTGTATGACTTGG	CAATATCCACCGTTAGAGAAC	7681
7984	AGATGTATGCTGAAGAACGTG	GGATCGTCAACCGTTAGTG	5109
8486	CACAGTGTGTTCGCCGGAAG	ACAGACAATGCACATCCTCTG	4773
8690	AGGTTGATACGGCAACGAG	GCAATCATTAGCATACAGATG	3326
8690INT	TGAGGAATCACGTGTTTG	ATCGAATCTATCCATGAAGATG	1746
90R3	CATCATTGTTATCGCTTTCAC	GCAACCGCAGTTCCTGTT	4912
R395	GCGGTACCCACTGATCTT	AGTCTACCAGGCATTCCGT	6250
95100	GAGGAGGGTCTGTCTAGATGT	TCGGAGATATCATAATCTGCGT	4031
100DR	TTATAGTTGCTCCCGAAAGC	AAGAAGATGCGGTTGTCTTG	2358

**Table 2:** Comparison of Illumina resequence human betaherpesvirus 6 (HHV-6A) U1102 to Reference sequence NC_001664.

ORF or Locus	Refseq bp	Refseq:SNP	Non-Synonymous SNP Amino Acid Change, Codons and (Reverse Complement Codons)	This Study or as Cited
DR-L DR6	6663	G:GG		
“	6665	G:GG	Frameshift extension	[16]
DR-L repeat	7623	G:C	“	“
Repeats left end U	8262	AGGAG:A		
“	8362	C:CGCG		
“	8518	T:C		
“	8559	ACAACA:CAC		
“	8591	AT:A		
“	8602	GG:G		
U10	18884	C:Indel1	Frameshift extension	[34,35]
U40	63871	G:T	Thr:His (ACG:CAG)	
“	63872	T:G	“	
U42	70446	A:ATC	HisCys:GlnLeuAsp (CACTGT:CAACTGCATG)	Jones & Teo 1993 in Genbank L20954
“	70449	G:GT	“	Jones, M. in Genbank X92436
U57 mcp	92149	C:G	Lys:Asn ( AAG:AAC)	
U58 tbp	94068	A:G	Met:Val ATG:GTG	
U83 chemokine	123740	C:G	AspGlu:GluGln GACGAG:GAGCAG	[15]
“	123741	G:C	“	“
U86 IE2	(128132)	(Indel G)	Frameshift U86/U87	In RefSeq; [17]
R1 repeats	128325	C:T		
“	128443	C:T		
“	128914	G:A		
“	129917	A:ATCC		
R2 repeats	132312	GG:G		
R3 repeats	137831	C:G		
“	137832	G:C		
“	137906	C:G		
“	137907	G:C		
U100	148154	C:G		
“	148155	G:C		
U100 gQ1	149553	C:G	Arg:Ala (CGT: GCT)	
“	149554	G:C	“	
DR-R DR6	157898	G:GG	Frameshift extension	[16]
“	157900	G:GG	“	
DR-R repeat	158857	G:C		

ORF = open reading frame; SNP = single nucleotide polymorphisms; Repetitive regions are shaded; Indel1: CACACCTATCGCGACCCTGTTTTATGTAGAAAGAATGACAAGCCAGAACAATGA; (“) indicates same as above.

**Table 3:** Minor variant polymorphism populations in HHV-6A U1102, passaged, and BACmid virus.

ORF or Loci	RefSeq bp	RefSeq :SNP	Amino Acid Change, Codons and (Reverse Complement Codons)	% New Reference	% BAC Virus	% Passaged Virus	Comment, Citations, and Other Strains with SNPs
DR	8016	A:C		-	87%	-	End T2
“	8077	C:CGA		96%	-	-	HHV-6A AJ KP257,584.1, GS KC465,951.1; CiHHV6A 2284 KT895,199.1, 1501 KT355,575.1; [37]; Sites cleavages after pac2; [26,31]
“	8080	G:GAC		-	86%	-	CiHHV6A 2284 KT895,199.1, 5055, 5814 [16,24]; “
Uleft repeat	8157	G:GC		-	-	78%	
“	8558	A:AC		88%	AAC:A 99%	90%	AC repeat
“	8561	AAC:A		-	100%	98%	HHV-6A AJ [15,31]
“	8601	CG:C		97%	87%	86%	
U2	8876	G:T	Ala314:Glu(GCG:GAG)	-	-	53%	
U19 UTR	28,371	C:CA		-	52%	72%	HHV-6A AJ
U31 tegument	45,667	A:T	Asn173:Ile AAC:ATC	-	73%	96%	
U33 capsid	52,148	A:G	Tyr330:His(TAT:CAT)	-	54%	69%	
U39 gB*	61,162	T:C	Thr193:Ala(ACG:GCG)	-	58%	99%	
U41 dbp	64,707	C:T	Ala972:Thr (GCT:ACT)	-	-	90%	HHV-6A GS KJ123,690.1
ORI repeat	67,674	T:C		-	-	54%	
“	67,860	T:C		-	-	34%	HHV-6A GS KJ123,690.1 & KC465,951.1
U50 capsid	81,583	G:A	Ala258:Thr(GCA:ACA)	-	59%	78%	HHV-6A GS KC465,951.1
“	82,158	G:C		-	67%	-	
U73 obp	109,214	G:A	Ser297:Asn(AGT:AAT)	-	-	99%	
U83 chemokine	123,504	G:GTT	f/s N-terminal extension	<1%	-	-	cDNA clone [13,14,15]; HHV-6A AJ KP257584; CiHHV6A: 5055 AIX09949, 23,090 AIX09956, 1501 KT355,575
U86 IE2	126,126	T:C	Asn395:Asp(AAT:GAT)	-	-	99%	
“	127,092	C:CTGA	Ser976:SerSer (TCA repeat ×9:×10)	-	45%	-	2 Ser insertion CiHHV6A 5055; 5814; [24]
R2 repeat	131,092	C:T		-	-	56%	IE2 intron
“	131,743	G:C		-	-	52%	“
“	131,745	A:C		-	-	62%	“
R2 region	132,303	TG:T		-	64%	56%	“ TGG:T HHV-6A AJ [31]
R3 repeat	140,872	G:A		-	96%	-	End 30× repeats from 137,775; latency IE1/2 intron [38]
U95	142,969	T:C	Trp10:Arg TGG:CGG	-	-	54%	
“	142,979	T:A	Ile13:Asn ATT:AAT	-	-	53%	
“	143,591	C:A	Ser217:Tyr TCC:TAC	-	-	76%	
U100 gQ2	146,729	C:T	C-terminal truncation; Trp186:stop (TGG:TGA)	<1%	-	-	[12]1 (in 7/8 gQ2 cDNA clones HHV-6A U1102)
“	146,862	G:A	Ser42:Leu (TCG:TTG)	-	60%	99%	Not in HHV-6A HHV-6A U1102 from [39]
U100 gQ1	149,251	Indel2:A	intron	-	44%	83%	HHV-6A AJ; CiHHV6A 2284, 5055, 5814 [24]

Indel2: AGGGGGGGG. Repetitive regions are shaded, DR repeats listed once here. * Adjacent to fusion loop 2 of Domain I (DI) homologue of human cytomegalovirus (HCMV) gB C246 [40], see text. (“) indicates same as above. (–) indicates no change in comparison to the reference genome sequence.

## Supplementary Materials

The following are available online at www.mdpi.com/1999-4915/10/1/16/s1, Figure S1: Alignment HHV-6A strain U1102 and HCMV strain Merlin gB ectodomains; Figure S2: The passage mutation in HHV-6 gB incrases interface hydrophobicity affecting subunit interactions; Figure S3: Multiple sequence alignment of HHV-6A gB with model templates from HCMV, HSV and EBV.
