# Supplementary material for: Analyses of Tissue Culture Adaptation of Human Herpesvirus-6A by Whole Genome Deep Sequencing Redefines the Reference Sequence and Identifies Virus Entry Complex Changes
Source: Viruses. 2017 Dec 31;10(1):16. doi: 10.3390/v10010016 (PMC5795429; doi:10.3390/v10010016)
Supplement: Supplementary file 1 [file viruses-10-00016-s001.pdf]

```
HHV6A  38   FRICSIAKGTDLMRFDRDISCSPYKS-NAKMSEGFFIIYKTNIETYTFPVRTYKKELTFQ  96
            +R+CS+A+GTDL+RF+R+I C+  K  N  + EG  ++YK NI  +TF VR Y+K LTF+
HCMV   91   YRVCSMAQGTDLIRFERNIVCTSMKPINEDLDEGIMVVYKRNIVAHTFKVRVYQKVLTFR  150
               FL1
HHV6A  97   SSYRDVGVVYFLDRTVMGLAMPVYEANLVNSHAQCYSAVAMKRPDGTVFSAFHEDNNKNN  156
              SY  +   Y L      +A P++E + +NSH+QCYS+ +     GTVF A+H D+ +N
HCMV   151  RSYAYIHTTYLLGSNTEYVAPPMWEIHHINSHSQCYSSYSR-VIAGTVFVAYHRDSYENK  209
                                         FL2   Thr193Ala
HHV6A  157  TLNLFPLNFKSITNKRFITTKEPYFARGPLWLYSTSTSLNCIVTEATAKAKYPFSYFALT  216
            T+ L P ++ +  + R++T K+ + +RG  WLY  + +LNC+VT  TA++KYP+ +FA +
HCMV   210  TMQLMPDDYSNTHSTRYVTVKDQWHSRGSTWLYRETCNLNCMVTITTARSKYPYHFFATS  269

HHV6A  217  TGEIVEGSPFFNGSN--GKHFAEPLEKLTILENYTMIEDLMNGMNGATTLVRKIAFLEKA  274
            TG++V  SPF+NG+N    +F E  +K  I  NYT++ D     N A    R +AFLE+A
HCMV   270  TGDVVDISPFYNGTNRNASYFGENADKFFIFPNYTIVSDFG-RPNSALETHRLVAFLERA  328

HHV6A  275  DTLFSWEIKEENESVCMLKHWTTVTHGLRAETNETYHFISKELTAAFVAPKESLNLTDPK  394
            D++ SW+I++E    C L  W      +R+E  ++YHF S ++TA F++ K+ +N++D
HCMV   329  DSVISWDIQDEKNVTCQLTFWEASERTIRSEAEDSYHFSSAKMTATFLSKKQEVNMSDSA  388

HHV6A  395  QTCIKNEFEKIINEVYMSDYNDTYSMNGSYQIFKTTGDLILIWQPLVQKSLMFLEQGSEK  394
              C+++E    + +++ + YN TY   G+  +F+TTG L++ WQ + QKSL+ LE+ + +
HCMV   389  LDCVRDEAINKLQQIFNTSYNQTYEKYGNVSVFETTGGLVVFWQGIKQKSLVELERLANR  448

HHV6A  395  ----I---R--------RRRDVGDVKSRHDILYVQLQYLYDTLKDYINDALGNLAESWCL  439
                +   R            + +++S H+++Y QLQ+ YDTL+ YIN AL  +AE+WC+
HCMV   449  SSLNLTHNRTKRSTDGNNATHLSNMESVHNLVYAQLQFTYDTLRGYINRALAQIAEAWCV  508

HHV6A  440  DQKRTITMLHELSKISPSSIVSEVYGRPISAQLHGDVLAISKCIEVNQSSVQLHKSMRVV  499
            DQ+RT+ +  ELSKI+PS+I+S +Y +PI+A+  GDVL ++ C+ +NQ+SV++ + M V
HCMV   509  DQRRTLEVFKELSKINPSAILSAIYNKPIAARFMGDVLGLASCVTINQTSVKVLRDMNVK  568

HHV6A  500  DAKGVRSETMCYNRPLVTFSFVNSTPEVVPGQLGLDNEILLGDHRTEECEIPSTKIFLSG  559
            ++ G      CY+RP+V F+F NS+  V  GQLG DNEILLG+HRTEEC++PS KIF++G
HCMV   569  ESPG-----RCYSRPVVIFNFANSSY-VQYGQLGEDNEILLGNHRTEECQLPSLKIFIAG  622

HHV6A  560  NHAHVYTDYTHTNSTPIEDI  579
            N A+ Y DY       +  I
HCMV   623  NSAYEYVDYLFKRMIDLSSI  642
```

Supplement figure 1. Alignment using Clustal of HCMV strain Merlin gB ectodomain region 91-642 with HHV-6A strain U1102 gB region 38-579 showing 228/541(42%) identity and 362/541(65%) similarity (+), with Domain I, fusion domain, region 79-291 underlined, fusion loops FL1 and FL2 in bold, and passage mutation Thr193Ala shaded. Alignments by meta-method in M-Coffee ((Wallace, I. M.; O ' Sullivan, O.; Higgins, D. G.; Notredame, C. M ‐ Coffee: combining multiple sequence alignment methods with T ‐ Coffee. Nucleic Acids Res. 2006, 34, 1692–1699).

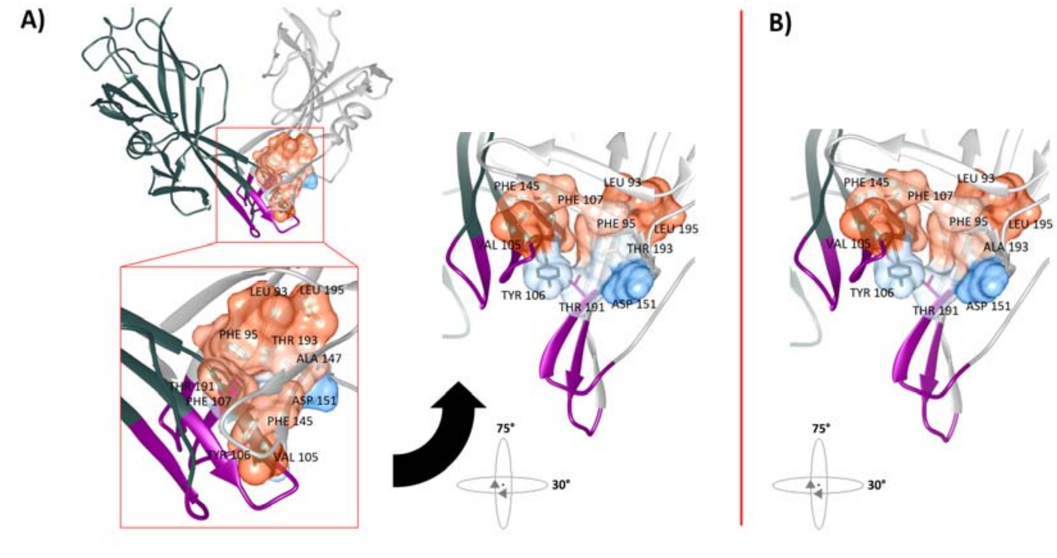

Supplement figure 2. The passage mutation in HHV-6 gB increases interface hydrophobicity affecting subunit interactions. Visualization of the residues at the interface between two subunits HHV-6A gB and residues surrounding the passage mutation site Thr193Ala. Two interacting subunits are in light and dark grey (A) and (B), with fusion loops, FL1 (residues 100-105) and FL2 (residues 184-191). The lateral chains for the residues 193, 93, 95, 145, 147, 151, 191, 195, 105, 106 and 107 located in the interface or near the position 193 were displayed (The residues 193, 93, 95, 145, 147, 151, 191 and 195 in one subunit and the residues 105, 106 and 107 in the other as visualising only one interface). The surfaces displayed correspond to their hydrophobicity (Kyte-Doolittle scale), blue hydrophilic to white at 0.0 to red hydrophobic. In (A) a global view of the interface between the Domains I of two subunits is shown (Top-left image) focusing on the subunit interfaces (Bottom left). The two other images in (A) correspond to a left-side view of the image located in bottom-left (Bottom-right image) and a top-view of the image in bottom right (Top-right image). The two images in (B) correspond to the same views with the passage substitution Thr193Ala.
Stability predictions between the two HHV-6A gB subunits show a stabilizing change of the free energy value of 0.056 for a Thr193Ala mutation in one of the subunits. This contributes to the hypothesis that stabilization of subunit-subunit interactions from mutation of Thr193 to the Ala residue acts via increasing the hydrophobicity of the hydrophobic pocket (Residues 93, 95, 145, 147 and 195 surrounding 193Thr, and all conserved in HCMV as shown above). These are in Domain I where the subunits interact (at the subunits interface; residues 193, 93, 95, 145, 147, 151, 191 and 195 in one face and residues 105, 106 and 107 in the other face).

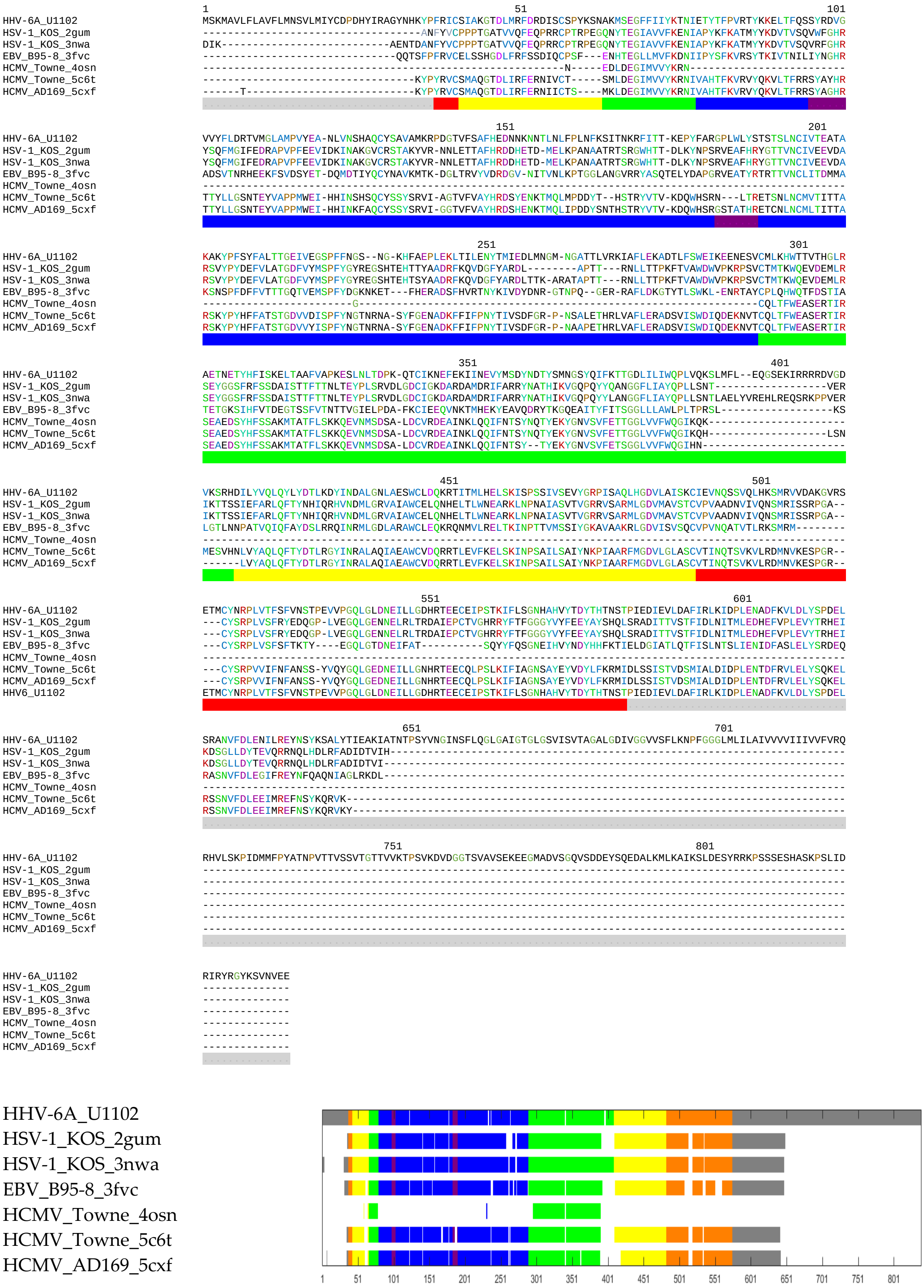

Supplement figure 3. Multiple sequence alignment of sequence of HHV-6A gB with model templates from HCMV, HSV and EBV. The colours correspond to domains I, II, III and IV with fusion loops in purple, as modelled in this study. Summary diagram showing the portions of sequences covered by the different template models (PDB ID : 2gum; 3nwa; 3fvc; 4oan; 5c6t and 5cxf) using meta-method for assembling multiple sequence alignments M-Coffee. (Wallace, I. M.; O'Sullivan, O.; Higgins, D. G.; Notredame, C. M-Coffee: combining multiple sequence alignment methods with T-Coffee. Nucleic Acids Res. 2006, 34, 1692–1699.)
